# Feasibility of a Culturally Adapted Dietary Weight-Loss Intervention among Ghanaian Migrants in Berlin, Germany: The ADAPT Study

**DOI:** 10.3390/ijerph18020510

**Published:** 2021-01-09

**Authors:** Stephen Amoah, Ruth Ennin, Karen Sagoe, Astrid Steinbrecher, Tobias Pischon, Frank P. Mockenhaupt, Ina Danquah

**Affiliations:** 1 Institute for Social Medicine, Epidemiology and Health Economics, Charité–Universitaetsmedizin Berlin, Corporate Member of Freie Universitaet Berlin, Humboldt-Universitaet zu Berlin, and Berlin Institute of Health, 10117 Berlin, Germany; ina.danquah@uni-heidelberg.de; 2 Institute of Tropical Medicine and International Health, Charité–Universitaetsmedizin Berlin, Corporate Member of Freie Universitaet Berlin, Humboldt-Universitaet zu Berlin, and Berlin Institute of Health, 13353 Berlin, Germany; enninruth@yahoo.com (R.E.); kaysagoe@yahoo.co.uk (K.S.); frank.mockenhaupt@charite.de (F.P.M.); 3 Molecular Epidemiology Research Group, Max Delbrück Center for Molecular Medicine in the Helmholtz Association (MDC), 13125 Berlin, Germany; asteinbrech@web.de (A.S.); tobias.pischon@mdc-berlin.de (T.P.); 4 Charité–Universitaetsmedizin Berlin, Corporate Member of Freie Universitaet Berlin, Humboldt-Universitaet zu Berlin, and Berlin Institute of Health, 10117 Berlin, Germany; 5 DZHK (German Centre for Cardiovascular Research), Partner Site Berlin, 10785 Berlin, Germany; 6 Department of Molecular Epidemiology, German Institute of Human Nutrition Potsdam-Rehbruecke (DIfE), 14558 Nuthetal, Germany; 7 Heidelberg Institute of Global Health, University Heidelberg, 69120 Heidelberg, Germany

**Keywords:** obesity, weight loss, diet, lifestyle, African migrants, Germany

## Abstract

Background: Dietary weight-loss interventions often fail among migrant populations. We investigated the practicability and acceptability of a culturally adapted dietary weight-loss intervention among Ghanaian migrants in Berlin. Methods: The national guidelines for the treatment of adiposity were adapted to the cultural characteristics of the target population, aiming at weight-loss of ≥2.5 kg in 3 months using food-based dietary recommendations. We invited 93 individuals of Ghanaian descent with overweight or obesity to participate in a 12-weeks intervention. The culturally adapted intervention included a Ghanaian dietician and research team, one session of dietary counselling, three home-based cooking sessions with focus on traditional Ghanaian foods, weekly smart-phone reminders, and monthly monitoring of diet and physical activity. We applied a 7-domains acceptability questionnaire and determined changes in anthropometric measures during clinic-based examinations at baseline and after the intervention. Results: Of the 93 invitees, five participants and four family volunteers completed the study. Reasons for non-participation were changed residence (13%), lack of time to attend examinations (10%), and no interest (9%); 64% did not want to give any reason. The intervention was highly accepted among the participants (mean range: 5.3–6.0 of a 6-points Likert scale). Over the 12 weeks, median weight-loss reached −0.6 kg (range: +0.5, −3.6 kg); the diet was rich in meats but low in convenience foods. The median contribution of fat to daily energy intake was 24% (range: 16–40%). Conclusions: Acceptance of our invitation to the intervention was poor but, once initiated, compliance was good. Assessment centers in the participants’ vicinity and early stakeholder involvement might facilitate improved acceptance of the invitation. A randomized controlled trial is required to determine the actual effects of the intervention.

## 1. Introduction

Adiposity is a growing public health problem, already affecting more than 2 billion adults worldwide. Of these, over 650 million individuals have obesity (body mass index (BMI) ≥ 30.0 kg/m^2^) [1]. Current projections indicate that by the year 2030, about 58% of the world’s adult population will have overweight or obesity (BMI ≥ 25.0 kg/m^2^) [2]. This will fuel the development of diabetes mellitus, cardiovascular disease, and cancers [3,4]. Among the growing group of sub-Saharan African migrant populations in Europe, overweight and obesity occur more frequently than in the European host populations [5,6]. Already, more than half a million people of African origin live in Germany [7] and their numbers are anticipated to increase rapidly [8]. Ghanaians form one of the largest groups of sub-Saharan African migrants in Europe [9,10]. In fact, around 46,000 Ghanaian migrants live in Germany [10,11] of whom 20% reside in Berlin [10]. General obesity (BMI ≥ 30.0 kg/m^2^) is prevalent in 14% of Ghanaian men and in 39% of Ghanaian women living in Berlin. For abdominal obesity (waist circumference > 102 cm for men and >88 cm for women), these figures are 15% in men and 71% in women [12].

Lifestyle modification constitutes the first-line treatment for obesity because it is safe and usually effective [13], but dietary interventions often fail among migrant populations because specific cultural needs are neglected [14]. Evidence from African Americans and Asian migrants in Europe emphasize the importance of cultural adaptations for weight-loss programs to produce better outcomes than generalized interventions [15,16,17]. These strategies may not be transferrable to West-African migrants in Europe because of their linguistic, educational and migration-related characteristics.

Given the obesity-related health problems and no previous interventions targeting migrants from sub-Saharan Africa in Germany, this feasibility study aimed at evaluating the practicability and the acceptability of a culturally adapted dietary weight-loss intervention among a group of well-characterized Ghanaian adults in Berlin. As a secondary objective, we aimed at exploring the weight-loss effect on changes in cardio-metabolic risk factors.

## 2. Materials and Methods

### 2.1. Study Population and Design

For the present ADAPT study (Feasibility of a Culturally Adapted Dietary Weight-Loss Intervention Among Ghanaian Migrants in Berlin), participants of the multi-center, cross-sectional Research on Obesity and Diabetes among African Migrants (RODAM) study [18] were re-invited in Berlin via telephone in September 2017. Owing to the well-established difficulties of enrolling migrant groups in population-based studies [12], we used documented contact details of previous RODAM participants in Berlin (*n* = 547) who were eligible (*n* = 93). Shopping vouchers (10 €) were offered for each completed examination visit as incentives to participate. In brief, the RODAM study was implemented between 2012 and 2015 and comprised Ghanaians aged 25–70 years living in rural and urban Ghana as well as in Amsterdam, Berlin and London. The study used standardized instruments for data collection at all the study sites, comprising questionnaire-based interviews, physical examination, and biological sample collection.

The inclusion criteria were BMI ≥ 25.0 kg/m^2^ or waist circumference >94 cm for men or >80 cm for women, Ghanaian migrant status (defined as being born in Ghana or having two parents born in Ghana), age ≥ 25 years, and the cooperation of the family cook or volunteer supporting the participant’s behavioral change. The exclusion criteria were known diabetes, receiving long-term oral corticosteroids or weight-loss medication, and current pregnancy.

### 2.2. Ethics Statement

The study protocol was reviewed and approved by the Ethics Committee of Charité-Universitaetsmedizin Berlin (EA1/151/17). The study was registered retrospectively at the German Registry for Clinical Trials (DRKS00013767). Prospective registration was not performed, because the study has not been planned as a randomized controlled trial. Therefore, the study was registered after the participants were recruited. The authors confirm that all ongoing and related trials for this intervention are registered. In order to finish the program before Christmas, the baseline assessments started on 2 October 2017, and the last visit was performed on 18 December 2017. All participants gave informed written consent prior to their enrolment.

### 2.3. Intervention Program

We adapted the guidelines for the treatment of adiposity by the German Society of Adiposity [19] (Table 1). To achieve behavioral changes at the individual level, we applied goal setting, behavioral contracting, and tailored health communication. These strategies were drawn from the Social Cognitive Theory of behavioral changes [20] and the stages of change construct of the Transtheoretical Model [21]. The adaptations of the treatment guidelines were based on the concept of Resnicow et al. [22], entailing an appropriate structure, process and strategy in adaptation.

Linguistic, constituent-involving and socio-cultural adaptations seem to be the most successful for weight-loss and dietary changes [15,16,23]. Therefore, we focused on the socio-cultural context and the languages of Ghanaians living in Berlin (Table 1). In this regard, trained personnel conducted questionnaire-based interviews in the participant’s preferred language, either English or a local Ghanaian language. The goal of this culturally adapted dietary intervention was to achieve weight-loss of at least 2.5 kg [24] which is based on international guidelines of a minimum 5% weight-loss of body weight during the 3-month period. The individual schedule for the study participants is shown in Table 2. In brief, the participants and their family volunteers received group counselling by an ethnically matched dietician. The counselling focused on the reduction of energy-dense foods, fewer eating occasions, and smaller portion sizes. Participants were encouraged to increase the consumption of fruits and vegetables. In addition, they received an information poster about a healthy Ghanaian diet and regular physical activity. The latter followed the recommendations of moderate to vigorous physical activity for at least 30 min per day. We organized monthly home-based cooking sessions with a dietician, and sent weekly smartphone reminders covering the participants’ dietary and activity goals. Lifestyle was monitored by culture-sensitive dietary assessment methods and by subjective and objective measurements of physical activity, respectively.

### 2.4. Recruitment

**Figure 1:**
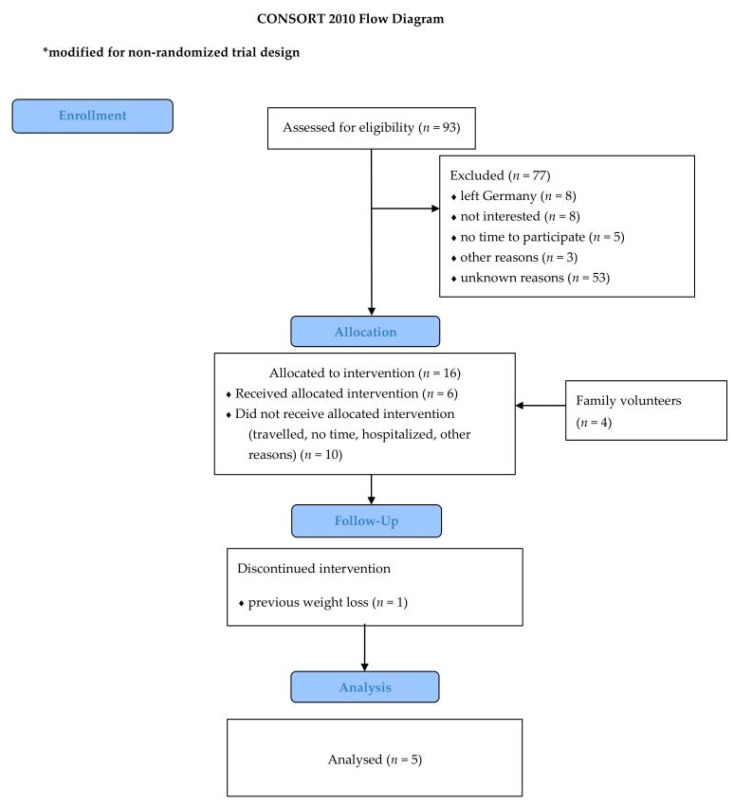
CONSORT Flow chart.

We used documented contact details of previous RODAM participants in Berlin (*n* = 547) [12] who fulfilled the inclusion criteria (*n* = 93). We invited them by phone. Reasons for non-participation were documented. Upon agreement, an appointment at the study center was scheduled for the baseline examination. The individual schedule for interviews and physical examinations is presented in Table 2. Figure 1 provides the CONSORT flow chart of the recruitment success. Of the 93 invited eligible participants, 16 were scheduled for the baseline examination. Finally, 6 individuals and 4 family volunteers were enrolled in the intervention study, translating into a participation rate of 6.5%.

### 2.5. Assessments of Demographics, Acceptability, and Lifestyle

Trained personnel conducted questionnaire-based interviews with the active study participants but not the family volunteers. These were performed in the participant’s preferred language, either English or a local Ghanaian language. Demographic characteristics included age and sex.

#### 2.5.1. Acceptability

The acceptability questionnaire was administered in weeks 7 and 12. We used a questionnaire that was based on the theoretical framework of acceptability, comprising seven component constructs [25]:

- Affective attitude: I enjoyed the diet and sports program;
- Burden: I easily integrated the diet and sports program in my daily life;
- Ethicality: The diet and sports program was important for me;
- Intervention coherence: I easily understood the diet and sports program;
- Opportunity costs: I am convinced by the diet and sports program;
- Perceived effectiveness: The diet and sports program will improve my health;
- Self-efficacy: The diet and sports program will help me to change my lifestyle.

There were six response categories to avoid the possibility of neutral answering, ranging from “strongly disagree” to “strongly agree”.

#### 2.5.2. Dietary Behavior

For information about the baseline habitual diet, we used data of the previous RODAM study, that had been collected with the semi-quantitative Ghana-Food Propensity Questionnaire (Ghana-FPQ) [26]. The Ghana-FPQ queries about the usual intake frequencies of food groups in predefined portion sizes during the preceding 12 months. It covers 134 items reflecting both indigenous Ghanaian and typical German foods. The German Nutrient Database (BLS) and the West African Food Composition Table were used to calculate the intakes of energy (kcal/d) and macronutrients (% of daily energy intake). During the course of the ADAPT study, 24-h dietary recalls (24HDRs) were conducted according to the 5-Steps Multiple Pass Method [27] at two points in time. Participants provided information on eating times, types of foods and beverages consumed in the past 24 h, and portion sizes. Specific information about brands and recipes were also recorded. Common Ghanaian household utensils were used to estimate the portion sizes.

#### 2.5.3. Physical Activity

Again, we used self-reported physical activity data of the previous RODAM study as our baseline information. Physical activity had been assessed by means of the WHO STEPwise approach to chronic disease risk factor Surveillance (STEPS) questionnaire [28]. The same tool was applied in the present ADAPT study in weeks 7 and 12. The STEPS questionnaire gathers information on physical activity in three settings (at work, travel to and from places and recreational) and sedentary behavior. Metabolic equivalents of task (MET)-hours were calculated. In addition to the self-reported physical activity data, we carried out objective measurements using a monitoring device (ActivPAL activity monitor; PAL Technologies Ltd., Glasgow, UK). This tool collects information about static and dynamic acceleration. The measurements were done for a week’s period on three different occasions. The lightweight device was worn discretely on the participants’ thigh for up to one week to quantify sedentary, upright and ambulatory activities as well as total MET-hours/day.

### 2.6. Physical Examinations

Similar to the questionnaire-based interviews, physical examinations were conducted only among the active study participants but not their family volunteers. A trained nutrition scientist conducted the physical examinations among participants in light clothes and without shoes. The measurements comprised body weight (kg; SECA 877), height (cm; SECA 217), waist circumference (cm) and hip circumference (cm) using a measuring tape. Systolic and diastolic blood pressures (mmHg) were measured (Boso Medicus Control; Bosch + Sohn GmbH, Jungingen, Germany) in triplicates after an appropriate resting time. The mean of the last two measurements was used for analysis.

### 2.7. Statistical Analysis

Baseline characteristics are presented as median and range for continuous variables and as percentage for categorical data. We calculated differences between baseline and follow-up data for secondary outcomes, i.e., weight-loss and lifestyle factors. All analyses were performed using Microsoft Excel 2016 (Microsoft Cooperation, Washington, DC, USA).

## 3. Results

### 3.1. Study Population

Two men and four women attended the ADAPT baseline examination. Table 3 presents the characteristics of the participants of the year 2014 (RODAM Study) and September 2017 (ADAPT baseline examination). The median age at baseline was 51 years (range: 25–62 years). The median BMI was 29.9 kg/m^2^ (range: 23.3–35.1 kg/m^2^) and the median waist circumference was 98.3 cm (range: 86.0–100.0 cm).

### 3.2. Practicability and Acceptability

We contacted 93 eligible individuals by phone. As depicted in Figure 1, the main reasons for non-participation were change of residence (13%), lack of time to attend clinic-based examinations (as opposed to their nearest Ghanaian practitioner; 10%), or no interest (9%); 64% of the non-participants did not want to give reasons for their decision. After the baseline examination, one individual actively withdrew from the study, because the person reported previous weight-loss and did not want to lose more weight. Thus, the analytical sample for all follow-up assessments comprised 5 individuals.

**Figure 2:**
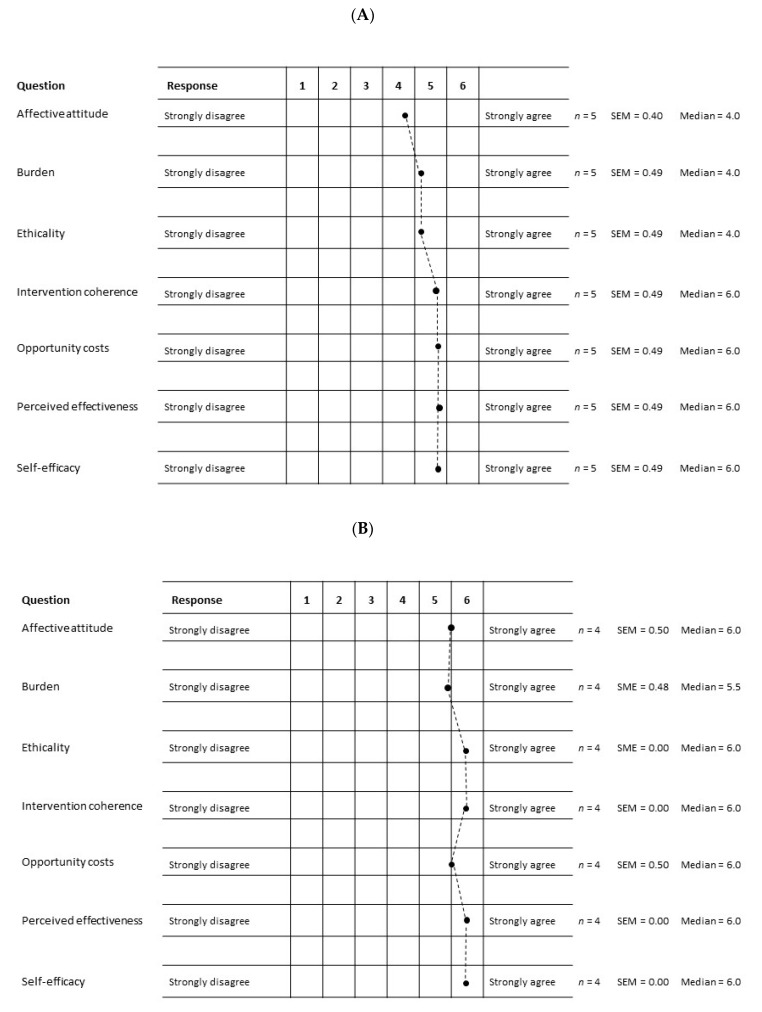
Acceptability of the culturally adapted dietary weight-loss intervention in week 7 (**A**) and in week 12 (**B**). Black dots indicate means. SEM, standard error of the mean.

Figure 2 shows the acceptability of the intervention programme according to the 7-items acceptability questionnaire in week 7 (Figure 2A) and week 12 (Figure 2B), using a 6-points Likert scale. In week 7, intervention coherence, opportunity costs, and self-efficacy reached the maximum score points, followed by perceived effectiveness (5.0), affective attitude (4.0), burden (4.0), and ethicality (4.0). These figures further improved until week 12 (Figure 2B).

### 3.3. Weight-Loss and Lifestyle Characteristics

The changes in anthropometric measures, lifestyle characteristics and clinical variables between baseline examination and follow-up are shown in Table 4.

After 12 weeks, the median weight-loss was −0.6 kg (range: +0.5; −3.6 kg), and median BMI and median waist circumference tended to be lower (Table 4). For lifestyle characteristics, the RODAM data served as the baseline information: median energy intake had been 2384 kcal/d (range: 992, 3361 kcal/d), and median energy expenditure had been 195 MET-h/week (range: 0–392 MET-h/week) (Table 3). The median difference in energy intake between these baseline data and the ADAPT follow-up information was −1480 kcal/d (range: −3330, −127 kcal/d). For physical activity, the median difference was 65 MET-h/week (range: −24, 249 MET-h/week) (Table 4).

**Figure 3:**
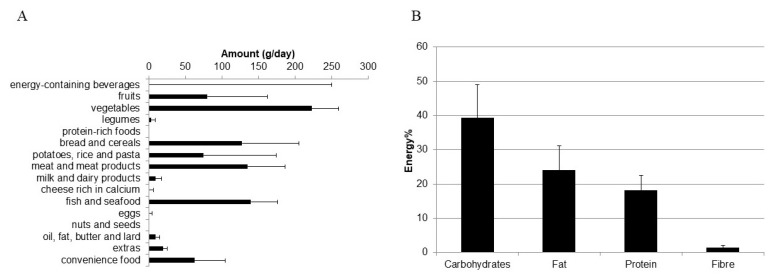
Median intakes of food groups (g/d) (**A**) and macronutrients (energy %) (**B**), based on the means of two 24-h dietary recalls. Error bars represent standard errors of the mean (SEM).

Figure 3 presents the food group consumption and macronutrient intakes during study conduct. In the 3-months intervention period, the dominating food groups were carbohydrate-rich items (bread, cereals, potatoes, rice and pasta), vegetables, meat and fish. The participants rarely consumed fruits and convenience foods, and never consumed energy-containing beverages (Figure 3A). Carbohydrates, fat and protein contributed each 39%, 24% and 18% to daily energy intake (Figure 3B).

**Figure 4:**
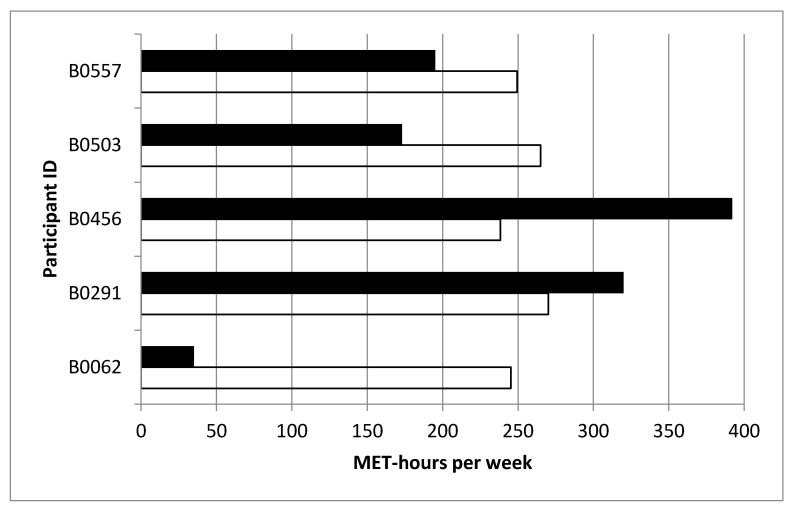
Median physical activity of weeks 7 and 12 for each ADAPT Study participant (participants codes, *y*-axis) by questionnaire-based self-report (black bars) and by objective measurement (white bars) (MET-hours per week).

Figure 4 presents the individual physical activity of the participants during the course of the ADAPT study, based on self-report and by objective measurements. The median self-reported energy expenditure was 144 MET-h/week (range: 20, 478 MET-h/week), while the median energy expenditure by ActivPAL was 249 MET-h/week (range: 238, 270 MET-h/week).

## 4. Discussion

This feasibility study examined the practicability and the acceptability of a 12-weeks culturally adapted, dietary weight-loss intervention among Ghanaian migrants with overweight in Berlin. Our study was the first attempt in Germany to provide a culturally adapted lifestyle intervention for West-African migrants. Innovative adaptations comprised linguistic, constituent-involving and socio-cultural components: a Ghanaian research team and an ethnically matched dietician to facilitate culturally appropriate communication and culinary knowledge to deliver nutrition education, respectively. Nudging, through regular text and image messaging, enhanced self-empowering and frequent assessments of dietary behavior and physical activity during the intensive intervention period facilitated self-contracting to achieve the individual weight-loss goals.

### 4.1. Practicability and Acceptability

For ethnic minorities in the United States, Nierkens et al. have reported response rates ranging from 31% to 97% for culturally adapted interventions aiming at smoking cessation, diet, and physical activity [16]. The lower participation rate of 6.5% in the present study was similar to the one seen in the RODAM baseline recruitment in 2015 [12], and may be attributed to mistrust and competing demands, as indicated in other African American population groups, too [29]. Indeed, in the previous RODAM Study, we experienced initial response rates of less than 5% following written invitation. In the ADAPT study, 19% of the contacted individuals claimed to have no time or no interest for such a program. This was also seen among Asian migrant populations in the UK, where community-orientated personal approaches for recruitment were most successful (83% response rate) [30]. Also, early sensitization and involvement of community leaders have contributed to enhanced enrolment of African migrants into health interventions in the Netherlands and in the UK [31,32]. Therefore, the present study has built on the documented contacts from former RODAM participants in Berlin.

Still, this target population appears to be highly mobile as indicated by the proportions of individuals who moved (8/93) or traveled (3/93). The poor intervention uptake may also stem from unawareness in the target population for adiposity as a risk factor of chronic diseases. In fact, in a qualitative study with Ghanaian migrants in Amsterdam (*n* = 46), few respondents associated hypertension with adiposity, even though many had overweight [33]. Moreover, Ghanaian adults perceive a certain degree of overweight as a sign of wealth, fertility, and beauty, particularly for and among women [34]. From this perspective, there seems to be no evidential need to engage in weight-loss activities. Lastly, food choices are not only influenced by individual factors, such as biological, demographic, psychosocial and situational aspects. Rather, interpersonal, environmental and political determinants take a growing role in the decision for food [35]. This system’s pressure might have generated reservations in the Ghanaian community about participating in the offered dietary intervention.

With regard to mistrust, this may be manifest regarding the German health system and its actors. Study participants rather preferred Ghanaian practitioners as study physicians. Therefore, future intervention studies aiming at dietary weight-loss among Ghanaian migrants should focus on early stakeholder involvement and evidential communication to create trust. In addition, we need to offer low-threshold interventions that minimize the time, the costs, and the potential of mistrust in the participants by employing ethnically matched practitioners, nurses and dieticians.

Notably, the intervention program was rated as highly convenient by those individuals who completed the program. While this could indicate selection of the most motivated people, it may also signal the cultural acceptability of our adapted program.

### 4.2. Weight-Loss and Lifestyle

The present feasibility study indicates that the culturally adapted dietary intervention may reduce body weight, BMI and waist circumference in this population over a period of 12 weeks, although the study lacks a comparison group. This corroborates previous findings that culturally tailored and facilitated interventions produce better outcomes than generalized interventions [15,16,17,36]. For instance, a tailored study among African Americans showed that body weight was reduced in the intervention group (mean difference: −2 ± 3.2 kg), but not in the standard care group (mean difference: 0.20 ± 2.9 kg, *p* = 0.02) over a period of 6 months [15]. To establish the actual health effects of the present intervention, a larger study with a comparison group is definitely required.

Regarding the dietary behavior among Ghanaian migrants, core food items of the traditional diet are maintained even several years after migration, including starchy roots and tubers, bread, rice, and leafy vegetables [26]. These typical dietary habits are still seen during the present intervention, and also cover low intakes of health-beneficial fruits and dairy products. This indicates that intensified efforts are required that go beyond the implemented dietary modifications, aiming at meals to support the integration of new, healthy food groups, paralleling the reduction of portion sizes for a negative energy balance. Also, there were hardly any changes to physical activity, indicating that additional promotion of regular physical activity is required.

### 4.3. Limitations

Since the study did not aim to test an effect of the intervention, no sample size calculations were performed. Owing to the small sample size, which impairs external validity of our findings, we refrained from concluding any effects of the intervention regarding biomedical data. The lack of a comparison group limits the interpretation of our acceptability results, because the poor participation rate could either result from the intervention program per se or from the reported reasons. Selection bias might have occurred, if study participants differ from non-participants in unobserved characteristics. The acceptability questionnaire used in this study may involve subjectivity bias in which the participants might have different interpretations of items resulting in different endorsements of ratings. For the assessment of dietary intake, detailed information about eating times, portion sizes, recipes and their preparations were gathered by culture-specific assessment tools [26]. However, two 24HDRs may not have captured day-to-day and weekend-to-weekday variations of food consumption. Different instruments were used during the RODAM study and the ADAPT project, which also complicated the comparison of energy intakes and macronutrients consumption.

## 5. Conclusions

This feasibility study examined the practicability and the acceptability of a 12-weeks culturally adapted, dietary weight-loss intervention among Ghanaian migrants with overweight in Berlin. The study showed a low participation rate, but 5 out of 6 enrolled participants completed the intervention and rated the program as highly convenient. The dietary behavior during the intervention period still relied on starchy foods and animal-based products and was low in fruit consumption. Yet, the proportions of consumed vegetables and the contributions of macronutrients to energy intake adhered to international dietary guidelines.

The present culturally adapted dietary weight-loss intervention for Ghanaian migrants in Germany has the potential to reduce adiposity and, thus, to prevent cardio-metabolic conditions in this vulnerable population group. In future studies, early stakeholder involvement, advocacy by community leaders, and sensitization of the Ghanaian community prior to the implementation are key factors for the success of this program. For the establishment of the actual weight-loss and its cardio-metabolic effects, a randomized, controlled trial is required.

## Figures and Tables

**Table 1:** Adaptations of the guidelines for the treatment of adiposity by the German Society of Adiposity (DAG).

Variable	German Society of Adiposity (DAG) Guidelines	ADAPT Intervention	Reasons for Adaptation
Participants	Individuals with adiposity	Ghanaian adult migrants (defined as born in Ghana or both parents born in Ghana) with either general overweight/obesity or abdominal overweight/obesity and one adult family volunteer. Main cook agrees to co-operate.	Recruit Ghanaian migrants with high prevalence rates of adiposity; encourage support from family members, particularly from those who are responsible for the family meals; encourage healthier lifestyle in the entire family
Inclusion criteria	Body mass index (BMI) ≥ 30.0 kg/m^2^ or waist circumference ≥ 88 cm for women and ≥102 cm for men, if BMI 25.0 < 30.0 kg/m^2^	Body mass index (BMI) ≥ 30.0 kg/m^2^ or waist circumference ≥ 88 cm for women and ≥102 cm for men, if BMI 25.0 < 30.0 kg/m^2^	Potential recruits may have central obesity, but have a low BMI; acknowledge the important role of central body fat accumulation
Setting	General practitioner	Community for recruitment, ethnically matched practitioner for examination, home setting for intervention	Encourage community and family involvement; increase compliance; reduce attrition
Duration of the intervention	3 months	3 months intensive intervention period with 1 group contact, 3 family-based contacts and weekly mobile phone reminders	Facilitate motivation, compliance, self-efficacy, family involvement, and sustainability
Weight loss goal	≥5% of initial body weight, if BMI 25.0 < 30.0 kg/m^2^; ≥10% of initial body weight, if BMI ≥ 30.0 kg/m^2^	≥2.5 kg in the intervention group	Realistic for Ghanaian migrants and still relevant to improve the cardio-metabolic profile
Physical activity (PA)	>30 min/day (≈ 1200–1800 kcal/week); mainly endurance sports; for individuals with BMI ≥ 30.0 kg/m^2^, increase PA in daily routine (e.g., walking, taking stairs); PA counselling: health-beneficial effects of physical activity beyond weight loss and PA goal setting	>30 min/day (≈ 1200–1800 kcal/week); increase PA in daily routine (e.g., brisk walking, taking stairs); Group counselling and lifestyle poster: health-beneficial effects of physical activity beyond weight loss; PA goal setting: pedometer; PA self-contracting: weekly mobile phone text messages	Most relevant; achievable recommendations, accounting for work load and family time; encouragement of self-chosen outdoor or gym activity in a group or alone; incorporates goal setting, behavioral contracting, and tailored health communication
Dietary intervention and targets	Dietary advice by general practitioner: daily energy deficit of 500 kcal; reduction of total fat and/or reduction of carbohydrates	Group counselling, lifestyle poster: reduced energy intake, not specific in nutrients; consultation with a dietician in the language of choice (German, English, local Ghanaian); reducing the intakes of frequently consumed foods that are rich in fats and carbohydrates; 3 home-based cooking sessions focusing on cooking methods, portion sizes, food choices, and fat amount for cooking; diet goal setting: 24-h dietary recall protocols; diet self-contracting: weekly mobile phone text messages	Bilingual dietary counselling available; Achievable and comprehensible approach, given the low level of formal education and health literacy in the study population; Engage the available family in a domestic setting especially those who prepare the family meals; Incorporates goal setting, behavioral contracting, and tailored health communication

**Table 2:** Individual intervention and examination schedule of the ADAPT study.

Week	1	2	3	4	5	6	7	8	9	10	11	12
Examinations												
Anthropometry	X											X
Oral glucose tolerance test	X											X
Blood pressure	X											X
Laboratory analyses												
Blood glucose (0, 30, 120 min)	X											X
C-Peptide, Insulin (0, 30, 120 min)	X											X
HbA1c	X											X
Fasting blood lipids	X											X
Intervention												
Group counselling	X											
Info poster	X											
Cooking session			X				X				X	
Smartphone reminder			X	X	X	X	X	X	X	X	X	X
ActivPAL set			X				X				X	
ActivPAL collect				X				X				X
24 h dietary recall								X				X
WHO STEPS activity questionnaire								X				X
Acceptability questionnaire							X					X

X represents the week in which an activity was undertaken.

**Table 3:** Baseline characteristics of the participants of the ADAPT feasibility study.

Characteristics	2014		2017	
	Median/Percentage	Range/Number	Median/Percentage	Range/Number
*n*	100%	6	100%	6
Age (years)	47.5	22.0–58.0	50.6	25.0–61.5
Sex (male)	33.3%	2	33.3%	2
Weight (kg)	75.5	64.0–83.9	77.4	62.8–87.6
Body mass index (kg/m^2^)	29.7	25.7–31.3	29.9	23.3–35.1
Waist circumference (cm)	92.2	83.1–105.1	98.3	86.0–100.0
Physical activity (MET-h/week)	195	0.0–392		
Energy intake (kcal/d) *	2384	922–3361		

* Energy intake was calculated based on the Ghana Food Propensity Questionnaire (Ghana). MET, metabolic equivalents of task.

**Table 4:** Differences in anthropometric and lifestyle characteristics between the ADAPT baseline and follow-up.

Characteristics.	Median/Percentage	Range/Number
Anthropometry		
∆ weight (kg)	−0.6	0.5, −3.6
∆ body mass index (kg/m^2^)	−0.3	0.2, −1.2
∆ waist circumference (cm)	−1.3	4.1, −4.5
Lifestyle characteristics		
∆ physical activity (MET-h/week)	65	−24, 249
∆ energy intake (kcal/d) *	−1480	−3300, −127

* Energy intake at baseline was measured by the Ghana Food Propensity Questionnaire and at follow-up by 24-h dietary recall. ∆-Change in values.

## Data Availability

The data presented in this study are available on request from the corresponding author. The data are not publicly available due to information that can potentially reveal the participants’ identity.
